# A solid state source of photon triplets based on quantum dot molecules

**DOI:** 10.1038/ncomms15716

**Published:** 2017-06-12

**Authors:** Milad Khoshnegar, Tobias Huber, Ana Predojević, Dan Dalacu, Maximilian Prilmüller, Jean Lapointe, Xiaohua Wu, Philippe Tamarat, Brahim Lounis, Philip Poole, Gregor Weihs, Hamed Majedi

**Affiliations:** 1 Department of Electrical and Computer Engineering, University of Waterloo, Waterloo, Ontario, Canada N2L 3G1; 2 Institute for Quantum Computing, University of Waterloo, Waterloo, Ontario, Canada N2L 3G1; 3 Waterloo Institute for Nanotechnology, University of Waterloo, Waterloo, Ontario, Canada N2L 3G1; 4 Institut für Experimentalphysik, Universität Innsbruck, Technikerstr. 25, 6020 Innsbruck, Austria; 5 National Research Council of Canada, 1200 Montreal Road, Ottawa, Ontario, Canada K1A 0R6; 6 Université Bordeaux, LP2N Institut d'Optique and CNRS, Talence F-33405, France

## Abstract

Producing advanced quantum states of light is a priority in quantum information technologies. In this context, experimental realizations of multipartite photon states would enable improved tests of the foundations of quantum mechanics as well as implementations of complex quantum optical networks and protocols. It is favourable to directly generate these states using solid state systems, for simpler handling and the promise of reversible transfer of quantum information between stationary and flying qubits. Here we use the ground states of two optically active coupled quantum dots to directly produce photon triplets. The formation of a triexciton in these ground states leads to a triple cascade recombination and sequential emission of three photons with strong correlations. We record 65.62 photon triplets per minute under continuous-wave pumping, surpassing rates of earlier reported sources. Our structure and data pave the way towards implementing multipartite photon entanglement and multi-qubit readout schemes in solid state devices.

## Abstract (web summary)

Multipartite photon states are desirable in quantum information technology but their generation in optical systems is less efficient with poor scaling. Here the authors demonstrate time-ordered photon triplets from a quantum dot molecule in a direct generation process with increased efficiency.

With the rise of quantum technologies, generalized quantum key distribution protocols^1^,^2^,^3^ based on multipartite entangled states could be stepping stones towards realizing real-world quantum networks^4^. While remarkable progress has been made on creating single photons and entangled photon pairs, multipartite correlated photon states are usually produced in purely optical systems by postselection techniques or cascading, with extremely low efficiency and exponentially poor scaling^5^,^6^,^7^. The most widespread technique for generating multipartite photon correlations relies on spontaneous parametric down conversion (SPDC) with low conversion efficiency^8^,^9^,^10^,^11^ and restricted scalability, which limits its production rate and applications. Moreover, in order to generate multipartite correlated photons, most schemes based on SPDC use the interference of photon pairs created by independent Poissonian sources and post-select the favoured subset of output photon states^7^,^10^,^12^,^13^,^14^, which significantly adds to the probabilistic nature of the process and the uncorrelated background light.

In contrast, quantum dots offer the most practical route in building scalable quantum architectures and their efficiency reaches almost unity per excitation pulse, enabling high count rates. The ground state of a single quantum dot hosts at most two bright excitons^15^, a biexciton, which can be controlled coherently^16^ to produce correlated photon pairs in a so-called cascade recombination process. Thus the creation of multipartite photon correlations in a single quantum dot requires exploiting energetically higher shells and phonon-mediated processes under heavy pumping, which lead to inevitable dephasing, line broadening and poor photon correlation visibility^17^,^18^. The coupled *s* shells of a quantum dot molecule (QDM), however, render additional excitonic states suitable for increasing the number of correlated photons possibly using coherent schemes. The wavefunctions of photogenerated excitons localized in the QDM s shells are coupled via molecular hybridization and Coulomb interactions^19^, thus the radiative recombination of such molecular excitons will naturally prepare correlated photons. The hybridization of carrier wavefunctions in a QDM is a strong function of the interplay between dot composition and interdot spacing. Nanowire-embedded quantum dots offer controllable size and composition^20^, which enable engineering of the QDM interdot coupling and its spectral properties. In addition, the core-shell structure alleviates the propagation and extraction of the optical modes that carry photons^21^ and promises more efficient detection of the photons emitted from higher-order excitons, which is a requisite in our photon correlation measurements^22^.

In the following, we demonstrate the creation of photon triplets using a QDM positioned inside an epitaxially-grown photonic nanowire. The photoluminescence (PL) spectrum of our QDM shows two sets of resonances governed by the QDM material and size. We identify these resonances by conducting a series of power-dependent and time-resolved spectroscopy experiments along with magneto-photoluminescence and photon correlation measurements. We observe a clear bunching–antibunching pattern when the photon correlations between each pair of triexciton, biexciton and exciton resonances are measured, which implies the emission of a photon triplet through a triple cascade recombination process. Employing the molecular *s* shells of the QDM aids us in achieving a far better photon correlation visibility than previous attempts in single quantum dots^17^,^18^. The photon-triplet-emission rate is estimated by conducting triple coincidence experiments in both continuous-wave and pulsed excitation regimes, showing a remarkable improvement compared to the creation of triplets in SPDC-based schemes. A realization of photon triplets from a triexciton forming in a QDM serves as an elementary step for the direct generation of multiphoton entanglement, which has so far been limited to photon pairs in solid state systems^23^.

## Results

### QDM structure

**Figure 1:**
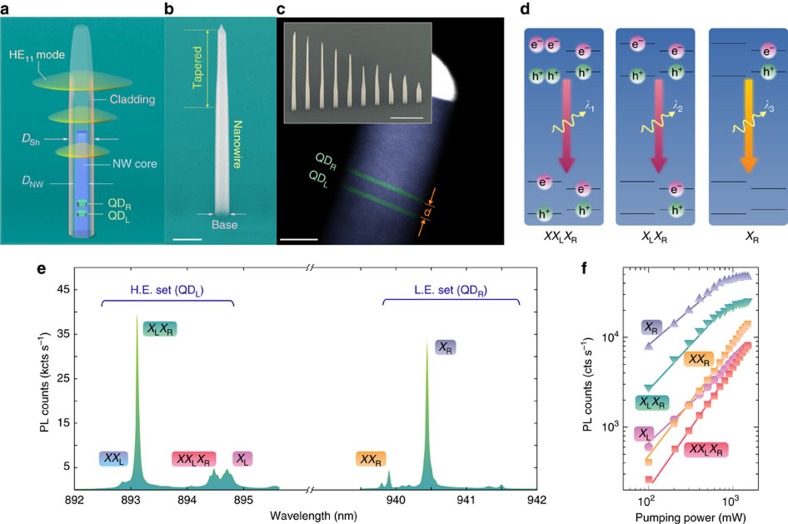
Structure and spectrum of a nanowire-QDM. (**a**) Schematic of a quantum dot molecule (QDM) embedded inside a clad nanowire. The best suited nanowires consist of a thin core region *D*_NW_=18–20 nm surrounded by a thick InP cladding (shell) *D*_Sh_=250 nm that waveguides at least one principal optical mode at QDM emission wavelengths ≈894 and ≈940 nm. (**b**) False-coloured scanning electron microscopy image of a spatially isolated nanowire with hexagonal crosssection incorporating a single QDM. The scale bar is 500 nm. (**c**) False-coloured transmission electron microscopy image of an InP nanowire (core) grown on (111)B substrate in wurtzite phase embedding two In(As)P quantum dots separated by ≈7 nm. The scale bar is 10 nm. Inset: The nanowires are site-controlled allowing excellent isolation of QDM spectrum from inhomogeneous broadening. The scale bar is 1 μm. (**d**) Triple sequential transitions: carrier configurations of high-energy (HE) triexciton, separated biexciton and low-energy (LE) exciton. (**e**) Optical spectrum of QDM comprising two prominent features at ≈894 and ≈940 nm. (**f**) photoluminescence intensity of the QDM resonances showing linear or superlinear dependence on the pump power.

Our QDM is composed of two InAs_*x*_P_1−*x*_ segments (*x*≈0.15 and 0.25) embedded inside an InP photonic nanowire that incorporates core and cladding regions^24^ (Fig. 1a). The thick cladding of 250 nm in diameter aids funnelling the QDM emission into the fundamental HE_11_ mode^25^ to be guided out toward the collection optics. The cladding is gently tapered (2°) at its apex to improve the photon extraction efficiency (Fig. 1b). The molecule contains two *h*_D_ ≈ 2.5–3 nm thick and *D*_NW_ ≈ 18 nm in diameter dots as confirmed by transmission electron microscopy imaging (Fig. 1c). The growth of the second dot QD_R_ is seemingly influenced by the strain field caused by the formation of the first dot QD_L_ during the molecular beam epitaxy process, giving rise to some compositional asymmetry of the molecule. Notice that even though the hybridization energy itself can exceed several tens of meV in strongly coupled double dots^19^, an important part of the *s*-shell splitting in the molecule studied here is induced by the above material composition change. Such an inherent asymmetry aids the localization of the heavy hole wavefunctions mainly inside the two individual dots rather than evenly spread throughout the molecule^26^. The similarity of dot and barrier compositions however leads to a comparatively weaker localization of the electron, and its orbital partially diffuses into the neighbouring dot. An interdot spacing of ≈8–10 nm was initially targeted in the vapour–liquid–solid growth mode; however, the arsenic tailing in our dots possibly reduces the effective separation *d* down to ≈7 nm. Considering the low arsenic concentration (0.15<*x*<0.25) of the dot segments, a thinner spacing would lift the barrier and aid the delocalization of electrons, or would promote the directional nonresonant tunnelling in the QDM^27^, whereas a larger spacing would impair the electron hybridization and interdot coupling. The yield of finding a suitable QDM in our investigated samples was 10%.

### Spectroscopy measurements and interdot coupling

In our experiment, the formation of a triexciton in the QDM entails the photogeneration of a biexciton (*XX*) in one quantum dot (QD_L_) along with an exciton (*X*) in the neighbouring dot (QD_R_) under continuous optical pumping. The predominant coupling mechanism among the two dots can be explained either via the wavefunction hybridization and Coulomb interactions^19^,^28^, or the direct energy transfer of excitons (Förster process)^29^, or nonresonant phonon-assisted tunnelling. The direct transfer of excitons is caused by long-range Coulomb interactions and typically occurs if the interdot energy splitting is small, at most a few meV. As shown later, the energy detuning of the constituent quantum dots is several tens of millielectronvolts in our molecule because of its structural asymmetry, hence the direct exciton transfer has a negligible impact on the interdot coupling here^29^,^30^. Moreover, the nonresonant tunnelling of carriers in QDMs is a function of the phonon spectral density, thus depends on the wavefunction overlap and particularly the energy difference of the states involved in the transition. This implies that any carrier tunnelling between the two detuned *s* shells of the constituent quantum dots in our QDM would require multiple acoustic phonon processes^31^. We will later demonstrate that nonresonant tunnelling plays a minor role in the interdot communication here, and therefore wavefunction correlation must be the primary source of coupling.

The studied QDM shows two distinguished high energy (HE) and low energy (LE) sets of spectral resonances at ≈894 nm and ≈940 nm corresponding to its molecular *s*-shell direct transitions (Fig. 1e). The formation probability of optically active indirect excitons should be small owing to the molecule asymmetry and rather single-dot-confined holes^32^. In addition to the conventional exciton (*X*_L_ or *X*_R_) and biexciton (*XX*_L_ or *XX*_R_) direct transitions belonging to QD_L_ and QD_R_, there exist energy-shifted biexciton and exciton transitions, *XX*_L_*X*_R_ at *λ*_1_=894.5 nm and *X*_L_*X*_R_ at *λ*_2_=893.1 nm emerging due to Coulomb interaction with *X*_R_ at *λ*_3_=940.9 nm. The carrier configuration related to the transitions creating the photon triplet is shown in Fig. 1d. They are assigned by acknowledging that bright interdot recombination is unlikely and that the *XX*_L_*X*_R_ and *X*_L_*X*_R_ resonances are located in the HE set. For simplicity, we name these two latter transitions triexciton and separated biexciton, respectively. The power-dependent PL intensities of the above resonances exhibit the expected linear and superlinear regimes for both series of regular and energy-shifted excitons and biexcitons, respectively (Fig. 1f). The emergence of *X*_R_ at the lowest excitation levels makes the conditional formation of separated biexciton and triexciton in QD_L_ more likely than that of *X*_L_ and *XX*_L_. *XX*_L_ grows on the shoulder of the neighbouring *X*_L_*X*_R_ resonance at higher excitation levels, which hinders resolving its power dependence over the entire range.

To understand the possible effect of nonresonant carrier tunnelling, we performed a time-resolved micro-PL experiment on the present QDM and another double dot, DD_2_, with identical single dot specifications, but an increased interdot spacing of over 30 nm to eliminate the coupling. The lifetime of the single exciton *X*_L_ of the QDM was measured at *τ*_d_=2.8±0.2 ns (a similar value can be inferred by comparing the *X*_L_*X*_R_ and *X*_R_ lifetimes as shown in Supplementary Note 4), whereas the *X*_L_ resonance of DD_2_ lasted *τ*_r_=2.5±0.2 ns. In general, the decay time *τ*_d_ of the exciton *X*_L_ in a molecule, where nonresonant tunnelling from QD_L_ to QD_R_ continuously takes place, is given by 1/*τ*_d_=1/*τ*_r_+1/*τ*_t_, where *τ*_r_ is the exciton radiative lifetime and 1/*τ*_t_ is the tunnelling rate. The fact that *τ*_d_ and *τ*_r_ are comparable within the accuracy of our experiment suggests that the impact of nonresonant tunnelling between the *s* shells of our QDM is negligible and perhaps a reverse mechanism exists between the *s* shell of QD_L_ and the nearby *d* shells of QD_R_ appearing at slightly higher energies in the spectrum. The nonresonant electron tunnelling is however enhanced at a small enough spacing (*d*<3 nm), where the barrier is lifted and QD_L_ is steadily emptied showing weak PL intensity (see Supplementary Note 2). The above observations indicate that the coupling in our QDM forms primarily via the hybridization of electron wavefunctions.

### Triple coincidence experiments

**Figure 2:**
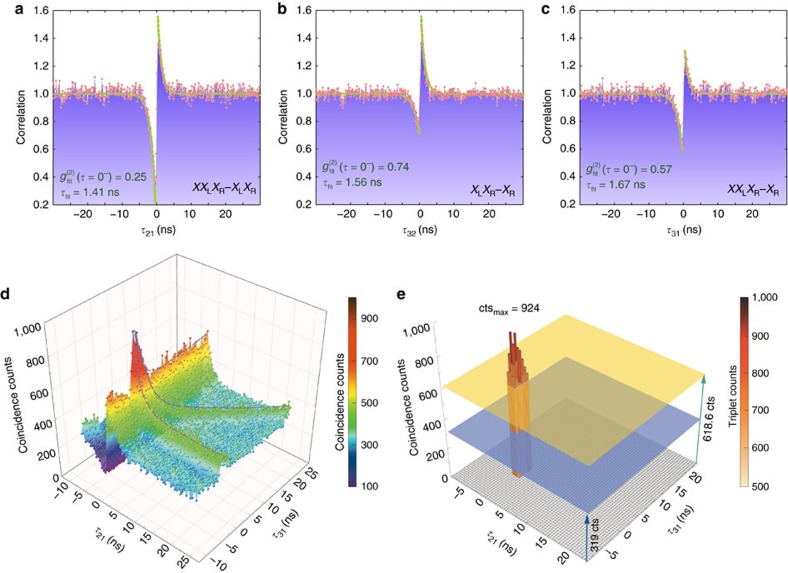
Dual-channel cross-correlations and triple coincidence histogram. (**a**–**c**) Normalized cross-correlations of *X*_R_, *X*_L_*X*_R_ and *XX*_L_*X*_R_ versus delay time measured at the excitation intensity of 6.9 W mm^−2^ showing a sequential triple cascade recombination. The antibunching dips are fitted with 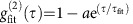 (*τ*<0), where the anticorrelation floor is limited by the background noise. (**d**) The triple coincidence histogram (total recording time 3 h) was measured at an intensity of 460 mW mm^−2^ and is plotted versus *τ*_21_ and *τ*_31_, linearly interpolated with a colour-mapped surface. The threefold coincidence peak near the origin signifies the strong temporal correlations of the emitted photons. (**e**) Events above the two-fold cascade threshold from **d** without interpolation plotted in 512 ps × 512 ps wide bins. The threshold level (yellow plane) was determined as the (peak) value of 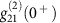 averaged over *t*_D3_ outside the triple coincidence window. For comparison the expected level of accidental triplet events is shown in blue.

The true character of *XX*_L_*X*_R_, *X*_L_*X*_R_ and *X*_R_ were fully identified by conducting a series of magneto-photoluminescence measurements to confirm that the Zeeman splitting of their spin fine structure comply with the theoretically predicted values, as presented in Supplementary Note 3. The next step was to measure the second-order autocorrelation function^33^ of each individual resonance and the cross-correlation functions^15^ 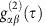 between various pairings (*α*, *β*) of distinct resonances (*τ*_*αβ*_=*t*_*Dα*_−*t*_*Dβ*_ denotes the delay time between photon detections by detectors Dα and Dβ). These correlations can provide insight into the coupling strength and the nature of the lines^34^. An autocorrelation experiment on every QDM resonance in our sample verified their low multiphoton emission probability by featuring an antibunching dip at *τ*=0. Among all possible cross-correlations of QDM resonances, the ones measured between *XX*_L_*X*_R_, *X*_L_*X*_R_ and *X*_R_ are of highest interest for the photon triplet characterization. A triplet state comprising temporally correlated photons *λ*_1_*λ*_2_*λ*_3_ originates from a triple sequential cascade in the QDM (Fig. 1d). In our correlation setup illustrated in Supplementary Note 1, a diffraction grating separates the *XX*_L_*X*_R_, *X*_L_*X*_R_ and *X*_R_ photons towards the detectors D1, D2 and D3. All cross-correlations 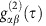 of the above three resonances feature an asymmetric bunching-antibunching behaviour^15^ as expected for cascade transitions (Fig. 2a–c). The cross-correlations between *XX*_L_*X*_R_ (or *X*_L_*X*_R_) and *X*_R_ are fitted with 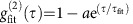 (*τ*<0) resulting in 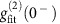
=0.71 (0.59), that is, considerably smaller than unity, which indicates that the system is indeed a molecule rather than two separate dots. Here, the non-zero level of correlation at *τ*=0^−^ can be explained by, first, the temporal dynamics of the transitions^34^, which depends on the ratio between their pumping rate *W*_p_ and decay rates Γ_*X*_ as further scrutinized in Supplementary Note 4 (increasing *W*_p_/Γ_*X*_ lifts the antibunching floor and suppresses the bunching peak); second, the parasitic background caused by the phonon sideband of the neighbouring weak spectral lines or stacking fault states. The effect of such background noise is more pronounced in cases where photons from the LE set contribute to the correlations, because they are collected by a fibre with a twice larger core that also collects more background emission (see Methods). Similarly, the cross-correlations between the remaining pairings of LE and HE resonances featured similar above antibunching characteristica, in contrast to the cross-correlations of our comparison double quantum dot DD2, which showed no signs of antibunching.

To prove that the QDM actually emits a photon triplet, we conducted a triple coincidence experiment^35^ by sending detector pulses D1 (as Start), D2 (as Stop1) and D3 (as Stop 2) into a time-tagging device. The time-resolved (512 ps wide bins) histogram versus *τ*_21_ and *τ*_31_ cointains the fully random contribution due to uncorrelated photons (319 counts) plus the sum (618.6 counts) of the three contributions that derive from two correlated photons and a third accidental one. We observe a large number of threefold coincidences in the vicinity of zero time delay (Fig. 2d) above the uncorrelated and partially correlated events. We recorded 20,744 photon counts in total (including 8,932 random background counts) integrated in 3 h in the coincidence window of *τ*_21_∈{−0.768, 1.28} ns and *τ*_31_∈{−1.28, 2.304} ns (see Fig. 2e). To ensure that the photon triplet generation rate is not overestimated, we subtract all the random or partially correlated events, which leaves us with 11,812 photon triplets corresponding to an average detection rate of 65.62 triplets per minute. We estimate that only 0.023% of all photon triplets could be detected because of the low detection efficiency of our detectors, *η*_D_=*η*_D1_ *η*_D2_ *η*_D3_ (*η*_D1_=25%, *η*_D2_=25% and *η*_D3_=15% at the respective wavelengths), along with non-ideal extraction efficiency *η*_C_=46% (see Methods), fibre coupling efficiency (*η*_F_=85%) and grating efficiency (*η*_G_=75%). The above photon triplet rate is, to the best of our knowledge, the highest recorded rate exceeding the values reported for direct generation of photon triplets via cascaded SPDC under continuous wave (cw) pumping^8^,^11^.

**Figure 3:**
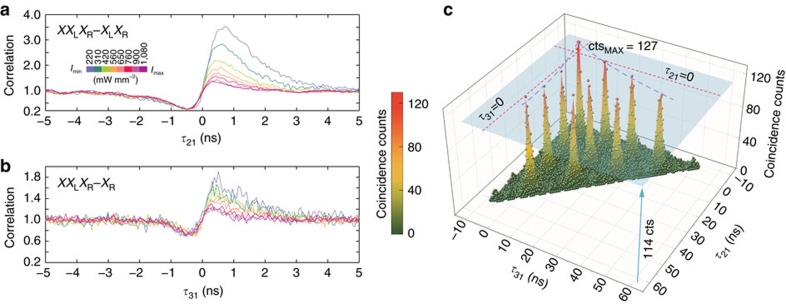
Bunching visibility and triple coincidence counts under pulsed excitation. (**a**,**b**) Normalized cross-correlations of the *XX*_L_*X*_R_ resonance with the *X*_L_*X*_R_ and *X*_R_ resonances measured at eight increasing power densities starting from 220 mW mm^−2^. The histograms are colour-coded according to the applied pumping levels. (**c**) The triple coincidence histogram measured in 80 min plotted versus *τ*_21_ and *τ*_31_ and linearly interpolated with a colour-mapped surface. The blue plane, at 114 counts, indicates the threshold level separating genuine photon triplet counts from the partially correlated photon counts.

In general, the bunching peak 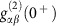 of a cascade decreases with the excitation rate, because the ratio of true cascade events versus individual excitations becomes less favourable, as has been observed in regular biexciton–exciton cascades of single quantum dots^36^. We examined this behaviour by applying increasing levels of pump power while recording the cross-correlations between the triexciton and the other two resonances (see Fig. 3a,b). The measurements were conducted in a regime where the PL intensity to background ratio hardly changed, thus the variation in the bunching peak was mainly a function of the ratio between the excitation rate *W*_p_ and transition lifetimes (1/Γ_*X*_). The difference between the bunching visibility of *XX*_L_*X*_R_−*X*_L_*X*_R_ and *XX*_L_*X*_R_−*X*_R_ cross-correlations in Fig. 3a,b also originates from the inequality of this ratio, *W*_p_/Γ_*X*_, in the *X*_L_*X*_R_ and *X*_R_ resonances, together with their unequal PL intensity measured by the silicon avalanche photodiodes at different wavelengths (see Methods). The suppression of the bunching visibility with increasing excitation power agrees with the results of our theoretical model based on the time propagation matrix method^34^, as explained in Supplementary Note 4, and reconfirms the cascaded nature of the selected transitions.

Finally, we demonstrated the formation of the triexciton and creation of photon triplets under pulsed excitation. For this purpose, the QDM was pumped non-resonantly with 2.6 ps pulses at 820 nm in the same cross-correlation setup used for the cw pumping regime (see Methods). Figure 3c illustrates the triple coincidence counts versus *τ*_21_ and *τ*_31_ measured in 80 min, featuring a central peak located at (*τ*_21_=0, *τ*_31_=0) and a 2D grid of side peaks with a temporal period of 12.5 ns, equal to the pulse cycle. The coincidence peaks in this histogram have contributions from fully and partially correlated events as previously identified in the cw regime. The central coincidence peak comprises all above contributions along with the fully correlated photon triplets occurring after the first excitation pulse, whereas the side peaks primarily result from the fully accidental and partially correlated events taking place between consecutive pulse excitations. We estimated the maximum number of partially correlated events at the side peaks to be 114 counts, and thus all the counts above this level and within a 5 ns 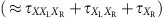 time window around the central peak were considered as true photon triplet counts, that is 363 photon triplets in 80 min (4.53 triplets per min). The lower rate of photon triplet generation here, as compared to the cw regime, could be attributed to the lower average cw-equivalent power, which essentially reduces the number of photogenerated carriers in the higher shells that eventually feed the ground state of the molecule within less than 1 ns. Moreover, in our method of calculation, the number of detection events considered genuine photon triplet counts is also a function of the ratio between the pulsed laser repetition rate and *τ*_*XX*_L___*X*_R__, *τ*_*X*_L___*X*_R__ or *τ*_*X*_R__, because longer lifetimes increase the probability of photon correlation between, e.g., *X*_R_ and re-excited *XX*_L_*X*_R_ or *X*_L_*X*_R_ from consecutive cycles. We predict that under coherent excitation, the background noise and the amplitude of the side peaks would drastically drop and the maximum triplet count rate would increase up to 17 kHz at the given efficiencies. Nevertheless, the above rate still tops the rates of direct photon triplet generation employing SPDC under pulsed pumping by an order of magnitude^37^.

## Discussion

Creating entangled photon triplets, as opposed to time correlated ones, remains as the next-step study goal to our present observations. The prospects of tripartite photon entanglement include, but are not limited to, multipartite quantum secret sharing, other quantum communication protocols^38^,^39^ and third party cryptography. As a relevant example, tripartite time-bin entanglement^40^ could be realized using the spin states of a triexciton bound in a QDM. Time-bin encoding has a clear benefit for long distance quantum communication through optical fibres because the relative phase between each two pulses with a few nanosecond temporal spacing is merely susceptible to a medium varying faster than this timescale. Implementing this kind of entanglement in a QDM, however, demands resonant pumping of the triexciton to encode the laser phase onto the emitted photon triplet in a relatively dephasing-free process^16^. In contrast to incoherent, pulsed excitation, almost a complete elimination of background light is expected under resonant pumping, and due to the absence of additional intraband relaxation processes the time jitter will be limited to the exciton radiative lifetime. In analogy with single quantum dots, coherent pulsed excitation of a QDM could prepare the triexciton in either of the singlet and triplet spin states, 0_*XX*,L_, *S*_R_ or 0_*XX*,L_, *T*_R_, where 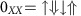, 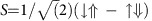 and 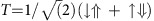, and {↑, ↓} ({⇑, ⇓}) denote the electron (heavy hole) spin localized in the left (L) or the right (R) quantum dot. 0_*XX*,L_, S_R_ (0_*XX*,L_, *T*_R_) could then decay to the {(*S*_L_, *S*_R_), (*T*_L_, *S*_R_)} ({(*S*_L_, *T*_R_), (*T*_L_, *T*_R_)}) biexciton states, followed by a second and eventually a third transition to 0_L_, *S*_R_ (0_L_, *T*_R_) and the ground state 0_L_, 0_R_ (see Supplementary Note 6 for the detailed diagram). These transition paths provide four sets of triple decays emitting three polarized photons H_1_H_2_H_3_, V_1_V_2_H_3_, H_1_H_2_V_3_ and V_1_V_2_V_3_ in the H and V linear basis, either of which could be utilized, for example, to create Greenberger–Horne–Zeilinger^10^ time-bin entangled photon triplets. Here, the coherent pumping of the triexciton is feasible through either employing three different coloured lasers in resonance with the transitions of interest or pumping virtual levels^16^. In either case, the output pulse of the lasers would be split into two pulses, early (*e*) and late (*l*). At sufficiently low pumping powers, a triexciton is formed by either the early or late pulse and the wavefunction of the three emitted photons can be represented as 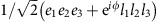. *φ* is the sum of the phases in the pumping interferometers, which will be added to the phases of the three analysing interferometers in a time-bin measurement.

At first glance, our rather low emission rate of photon triplets under the incoherent pulsed excitation regime might imply an inefficient generation of entangled photon triplets using QDMs. However, we predict a drastic improvement of the photon triplet counts under resonant excitation due to the profound suppression of background noise and accidental coincidences. In this case, the triplet generation rate is approximately given by 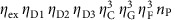, where *n*_P_ denotes the pulse repetition rate, and *η*_ex_ is the excitation probability of the triexciton, which can potentially reach up to 90% with an optimized pulse length as previously demonstrated for the biexciton^41^. Under such circumstances, improving the detection efficiency, for example, by employing near-ideal superconducting nanowire photodetectors^42^, or enhancing the light extraction efficiency, by embedding a reflective layer under the nanowire base^21^, could potentially boost the integrated triplet counts by two orders of magnitude.

In conclusion, we have demonstrated that a triexciton bound in a QDM can originate time-ordered photon triplets in a cascaded process. We expect to improve the triplet generation rate by reducing the inter-dot energy splitting, deterministic coherent pulsed excitation of the triexciton to reduce the background, and enhanced collection efficiency. Triple excitons forming in the *s* shells of a QDM should, in priciple, benefit from far better coherence properties than *p*-shell excitons in single quantum dots, because their coherence time *T*_2_ is not subject to the dephasing caused by the *p*-to-*s* phonon scattering relaxation. The necessity of populating higher shells in single quantum dots also requires strong optical pumping, which further adds to the spectral diffusion and the photon decoherence problem. The inhomogeneous broadening observed in our current QDM samples, however, arises from the stacking faults in the nanowire, which function as efficient charge traps and cause the spectral wandering^43^. The density of such stacking faults is expected to be reduced by improving the molecular beam epitaxy growth conditions at higher temperatures (500 °C) in the near future. With the earlier demonstration of quantum-dot-based quantum key distribution^44^, our device facilitates the implementation of multiparty quantum secret sharing on integrated semiconductor chips.

## Methods

### Nanowire-QDM fabrication

The InP nanowires with embedded In(As)P quantum dots are grown using selective-area vapour–liquid–solid epitaxy. The nanowires are grown on an SiO_2_-patterned (111)B InP substrate. The pattern consists of circular holes defined using electron-beam lithography and hydrofluoric acid wet-etch. A single gold particle is deposited in each hole using a self-aligned lift-off process, with the size of the particle determined by the hole size and the thickness of deposited gold. We employ chemical beam epitaxy with trimethylindium and pre-cracked PH_3_ and AsH_3_ sources. The growth temperature is 420 °C. Two growth modes are utilized to grow a nanowire core, which defines the quantum dots, and a shell, which defines the cladding of photonic nanowire. The nanowire core is grown under a reduced PH_3_ flow resulting in an untapered InP nanowire with a diameter corresponding to the gold catalyst particle, approximately 20 nm in this work. The nanowires are pure phase wurtzite with less than 1 stacking fault per micron^20^. The double In(As)P quantum dots are grown by switching the group V species from phosphorous to arsenic to grow the first dot, switching back to phosphorous to grow the InP spacer, then switching back to arsenic to grow the second dot while maintaining a constant flux of trimethylindium. Samples were grown with quantum dot growth times of 2.5 and 3 s, and with spacer times of 10, 15, and 60 s. The interdot spacing for a given growth time between dots depends on the core diameter due to a diameter-dependent growth rate^45^. By using a diameter-dependent growth model^45^ we calculate an interdot separation of 8–20 nm for core diameters of 18–24 nm. Details of the spacer-dependent interdot coupling are beyond the scope of this work and will be published elsewhere. The spacer of QDM studied here is 10 s (≈7–8 nm) that provides the optimum coupling. The nanowire shell is grown by increasing the PH_3_ flow rate by a factor of three, which reduces the indium adatom migration length and promotes deposition on the nanowire sidewall facets. The shell is grown to reach base diameters of 250 nm, resulting in nanowires with heights of ≈5 μm and tapers of ≈2°.

### Optical experiments

The sample is cooled down to 6 K in a customized and thermally stabilized liquid-helium continuous-flow cryostat. The QDM is nonresonantly excited either by a cw or a ps-pulsed Ti:Sapphire laser at 820 nm with 8.4 ps (or 2.6 ps for the cross-correlation measurement) pulse duration (80 MHz repetition rate) slightly above the wurtzite InP band gap 1.49 eV (832 nm) and the donor–acceptor recombination level 1.44 eV (861 nm). We excite the QDM via a separate objective rather than the collecting objective even though this is not reflected in the setup schematic in Supplementary Note 1. The molecule luminescence is collected using an objective lens with a numerical aperture of 0.7 and dispersed by grating monochromators with a spectral resolution of ∼0.01 nm to split the spectral lines and send the respective photons into separate avalanche photodiodes (APD). APDs are identical with ≈300 ps temporal resolution and ≈25% (≈15%) detection efficiency at 893 nm (940 nm). The combination of spectrometer and charge coupled device camera enables performing *in situ* spectroscopy during the recording of counts in the correlation measurement setup (composed of APDs and ps time-tagging module). Only two APDs register the photon counts to conduct the autocorrelation and the conventional dual-channel cross-correlation analysis, whereas all the three APDs are in use for the triple coincidence experiment. In the dual-channel correlation measurements, the HE set resonances are cross-correlated utilizing two 5 μm core optical fibres for photon collection. In the triple coincidence experiment, we collected from the LE set using a single mode fibre with 9 μm core optimized for the telecommunication wavelength, which operates as a multimode fibre at 940 nm. The multimode character improves the collection efficiency without the requirement for an optimized mode matching. However, owing to the small core radius the background light picked up from *X*_R_ is suppressed and the antibunching dip in the triple coincidence histogram is improved compared to a 125 μm core multimode fibre. To estimate the extraction efficiency of the nanowire, we calculate the probability of a biexciton-exciton coincidence 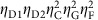 from the dual-channel cross-correlation histogram to be 0.54%, which yields *η*_C_=46%. To produce the power-dependent cross-correlation histograms in the cw excitation mode, we started from 220 mW mm^−2^ (with 4 μm spot size and the excitation objective tilted 22° from the optical table axis) and raised the pump power to linearly increase the indirect biexciton luminescence *XX*_L_*X*_R_. Therefore, the pump power scales up approximately in a quadratic fashion until the *X*_L_*X*_R_ resonance is saturated. To resolve the associated lifetimes, the QDM is heavily pumped within each pulse using the Ti:Sapphire laser in a way that its resulting spectrum exactly resembles the one under cw excitation. In the triple coincidence experiment under pulsed excitation, the pumping power was adjusted to 40 μW (with 2.6 ps pulse duration), which translates to a peak intensity of 905 mW mm^−2^. The temporal resolution of the detectors D1, D2 and D3 was set to 512 ps. The laser spot size was approximately 7.5 μm on the sample (measured perpendicular to the beam, which was aligned under an angle of approximately 50° with respect to the nanowire axis). For the magneto-optical measurements the setup remains unchanged except that the cryostat is replaced by a continuous flow exchange gas cryostat with a 7 T split-pair superconducting magnet. The QDM was excited co-linearly to the collection through the collection objective with a Ti:Sapphire laser. For the mixed Voigt-Faraday (tilted) configuration the sample was rotated 12° inside the cryostat.

### Data availability

The data that support the findings of this study are available from the corresponding author upon request.

## Additional information

**How to cite this article:** Khoshnegar, M. *et al*. A solid state source of photon triplets based on quantum dot molecules. *Nat. Commun.* **8,** 15716 doi: 10.1038/ncomms15716 (2017).

**Publisher's note**: Springer Nature remains neutral with regard to jurisdictional claims in published maps and institutional affiliations.

## Supplementary Material

Supplementary Information Supplementary Figures, Supplementary Table, Supplementary Notes and Supplementary References.
