# Supplementary material for: A solid state source of photon triplets based on quantum dot molecules
Source: Nat Commun. 2017 Jun 12;8:15716. doi: 10.1038/ncomms15716 (PMC5472777; doi:10.1038/ncomms15716)
Supplement: Supplementary Information — Supplementary Figures, Supplementary Table, Supplementary Notes and Supplementary References. [file ncomms15716-s1.pdf]

Supplementary Information

Supplementary note 1 We use either cw or pulsed Ti:Sapphire laser to pump the Quantum dot molecule (QDM) depending on the nature of experiment as described in Methods. The emission of cryogenically cooled nanowire-QDM is either sent to the spectroscopy module, composed of spectrometer and charge coupled device (CCD), or photon statistics module, composed of gratings for dispersing photons and correlation setup, including avalanche photodiodes (APDs) and time-tagging card, for recording the correlation histogram. To resolve triple coincidence counts, photons from $XX_{\mathrm{L}}X_{\mathrm{R}}$, $X_{\mathrm{L}}X_{\mathrm{R}}$ and X_{R} are sent to three identical APDs (D1-D3). The polarization module, composed of quarter wave plate (QWP), half wave plate (HWP) and polarizer (Pol) is employed to measure fine structure splittings.

Supplementary Note 2 We performed a photoluminescence (PL) spectroscopy measurement on an ensemble of nanowire-embedded QDMs. Low excitation powers were applied to avoid the activation of multiexciton complexes. The dependence of the exciton resonance associated with each dot, X_{R} and X_{L}, on the interdot spacing is plotted in Supplementary Figure 2. Since the spacing is a function of both the QDM diameter and the growth time (see Methods), the diameter-dependent shifts of the X_{R} and X_{L} resonances are removed in order to give better insight into the effect of spacing on hybridization. The spacing values shown here are nominal values d_{n} intended during the growth process and the effective interdot spacings d_{eff} are smaller as explained within the main text. At a constant pump power, we consistently noticed the decrease in the PL intensity of the high energy (H.E.) set (or X_{L}) by reducing the spacing until it almost disappeared for the

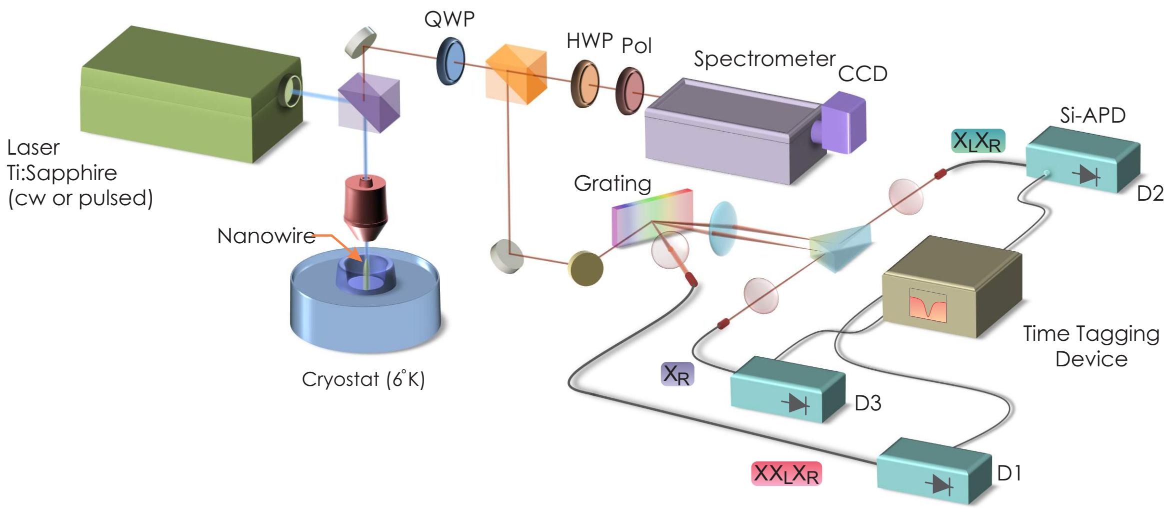

Supplementary Figure 1: Measurement setup Simplified optical setup used for the correlation measurements. Nanowire sample is cooled down to 6°K using a microscopy cryostat and optically pumped with a continuous wave (cw) or a pulsed laser as described in Methods. The polarization state of the emitted light from QDM is controlled by a quarter wave plate (QWP), a half wave plate (HWP) and a polarizer (Pol). The spectrum of the QDM is continuously monitored using the spectrometer and a charge coupled device (CCD). Single photons from each resonance are dispersed by a high-resolution grating and sent to three avalanche photodiodes (APDs) and a time tagging device to perform time correlation measurements.

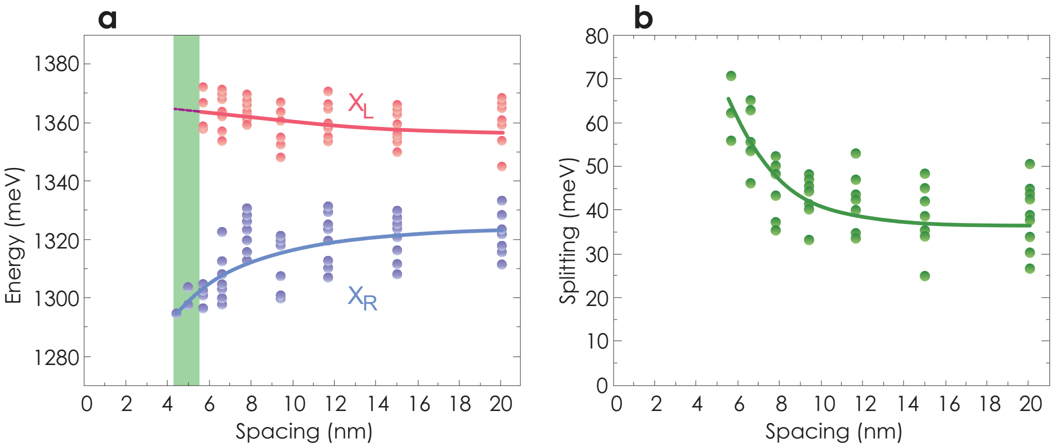

Supplementary Figure 2: QDM exciton energy (a) The evolution of the QDM exciton energies as a function of the nominal interdot spacing d_{n}. The green-shaded area determines where the H.E. photoluminescence intensity becomes weak. (b) Energy splitting of the H.E. and low energy (L.E.) sets versus the nominal interdot spacing.

nominal spacing of $d_{\mathrm{n}} \sim 5.5$ nm ($d_{\mathrm{eff}} \sim 3$ nm). This indicates the direct electron tunneling from the smaller dot into the larger dot occurs with tunneling rates higher than the exciton decay rate in the smaller dot at such interdot spacing. For a large spacing, for example $d_{\mathrm{n}} > 18$ nm, an energy separation of at most 50 meV exists between the two resonances, which originates from structural asymmetry. However, for smaller spacing $d_{\mathrm{n}} < 18$ nm, the energy separation increases, partly because of the stronger hybridization energy of the QDM. This proves that at the spacing of $d_{\mathrm{n}} \sim 10$ nm ($d_{\mathrm{eff}} \sim 7$ nm) (corresponding to the QDM studied in the main text), the wavefunction hybridization and Coulomb interactions result in the coupling of the two quantum dots.

Supplementary Note 3 We conducted magneto-optical experiments to identify the resonances by comparing their Zeeman splitting to the theoretically predicted values. A bright exciton such as X_{R} comprises a spin doublet $|S_{X_{\mathrm{R}},||}\rangle = \{|+1\rangle = |\downarrow\Uparrow\rangle, |-1\rangle = |\uparrow\Downarrow\rangle\}$ which splits by $\Delta_{X_{\mathrm{R}}} = g_{X_{\mathrm{R}},||}\,\mu_{\mathrm{B}}\,B_{||}$ in Faraday configuration (magnetic field $B_{||}$ applied along the nanowire axis), where

$\downarrow\uparrow$ ($\Downarrow\Uparrow$) represents the electron (heavy hole) spin, $g_{X_{\rm R},||} = g_{\rm e_R,||} + g_{\rm h_R,||}$ is the axial exciton g-factor and $\mu_{\rm B}$ is the Bohr magnetron. A regular biexciton has zero spin, thus its transition to the exciton state experiences the same splitting. The interdot biexciton $X_{\rm L}X_{\rm R}$ is however fourfold degenerate $|S_{X_{\rm L},||}, S_{X_{\rm R},||}\rangle = \{|\pm1,\pm1\rangle\}$ at $\mathbf{B} = 0$ with four bright decay channels down to the $X_{\rm R}$ doublet, $|1,\pm1\rangle \rightarrow |\pm1\rangle$ and $|-1,\pm1\rangle \rightarrow |\pm1\rangle$, if spin flip processes are ruled out.[1] These decay channels split into two doublets spaced by $\Delta_{X_{\rm L}X_{\rm R}} = g_{X_{\rm L},||}\,\mu_{\rm B}\,B_{||}$. Finally, the triexciton produces a bright spin doublet $|S_{XX_{\rm L},||}, S_{X_{\rm L}X_{\rm R},||}\rangle = \{|0,\pm1\rangle\}$ which decays via two doubly degenerate transitions to the separated biexciton, $|0,\pm1\rangle \rightarrow |1,\pm1\rangle$ and $|0,\pm1\rangle \rightarrow |-1,\pm1\rangle$, with $\Delta_{XX_{\rm L}X_{\rm R}} = g_{X_{\rm L},||}\,\mu_{\rm B}\,B_{||}$ energy splitting. We investigated the QDM PL under magnetic field in the Faraday configuraion (not shown here) and a tilted ($\theta = 12°$) configuration (Supplementary Figure 3a). The Zeeman coupling of the bright and dark spin states should cause a fourfold splitting of the fine structure in charged excitons should occur even at small magnetic fields in the tilted configuration, whereas the intrinsic dark-bright splitting in neutral excitons prohibits such mixing except for high field magnitudes.[2] Observation of dual splittings in the tilted configuration would therefore imply that the resonances do not originate from charged states. The PL intensity versus magnetic field B_θ and wavelength is shown in Supplementary Figure 3b, where the dashed lines indicate the evolution of the resonances. The H.E. set is displayed in higher pumping levels ($P_2 = 10P_1$) well above the onset of the triexciton emission. We fit the spectral peak displacement with $\pm g_{X,\theta}\,\mu_B\,B_\theta + \gamma_{X,\theta}\,B_\theta^2$ to remove the diamagnetic shift ($\propto B_\theta^2$) present in the weak-field regime,[3] and obtain $g_{X_{\rm R},\theta} = -0.92$ and $\gamma_{X_{\rm R},\theta} = 8.30\ \mu{\rm eV/T^2}$ for the L.E. exciton and $g_{X_{\rm L}X_{\rm R},\theta} = -1.62$ and $\gamma_{X_{\rm L}X_{\rm R},\theta} = 11.94\ \mu{\rm eV/T^2}$ for the separated biexciton. The spectra and corresponding Zeeman splittings of

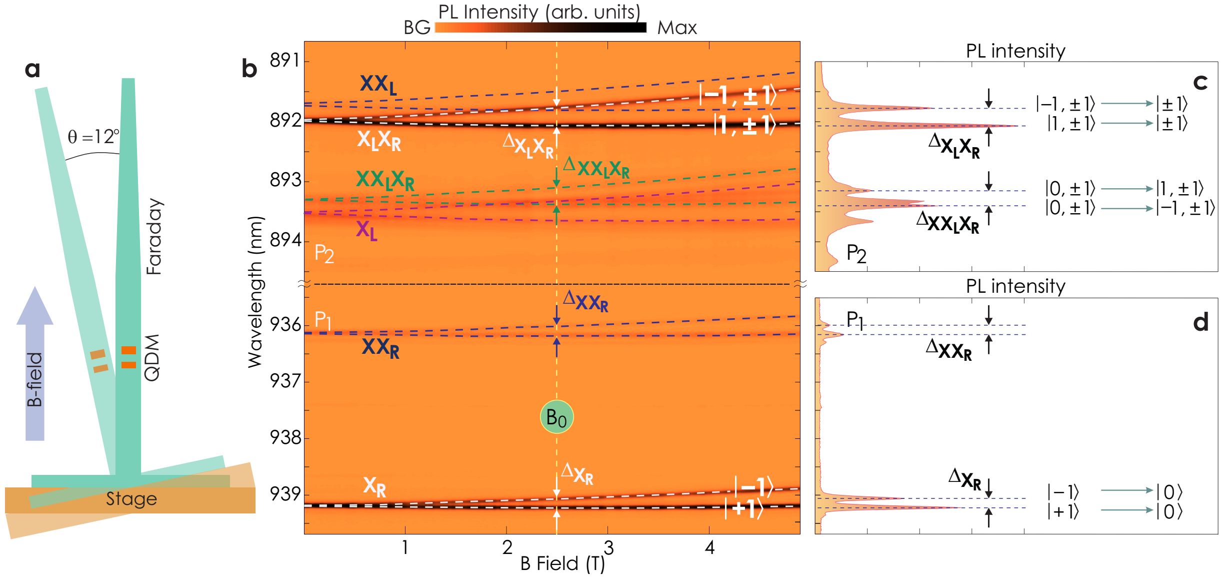

Supplementary Figure 3: QDM resonances versus magnetic field a) Schematic of nanowire-QDM in magnetic field in Faraday and tilted ($\theta = 12°$) orientations. (b) QDM PL intensity versus tilted magnetic field B_θ. The L.E. set is measured at $P_1 = 0.5\ \mu$W and shows two prominent doublets. The H.E. set is scanned at $P_2 = 10P_1$ where the triexciton spin splitting becomes visible with respect to background light. BG and Max stand for the background and maximum counts, respectively. a.u., arbitrary units. (c-d) Spectra of H.E. and L.E. sets measured at $B_0 = 2.5$ T as highlighted by the yellow dashed line in (b). The doublet and singlet transitions under investigation are labelled with their associated spin states for further clarity. The biexciton transitions in each set, either shifted or unshifted, mirror their corresponding exciton transitions, $\Delta_{XX_\mathrm{L}X_\mathrm{R}} = \Delta_{X_\mathrm{L}X_\mathrm{R}}$ $(= \Delta_{X_\mathrm{L}})$ and $\Delta_{XX_\mathrm{R}} = \Delta_{X_\mathrm{R}}$.

the $X_\mathrm{L}X_\mathrm{R}$ (and $XX_\mathrm{L}X_\mathrm{R}$) doublets along with X_R (and XX_R) singlets at $B_\theta = 2.5$ T are shown in Supplementary Figures 3c-d, verifying that $\Delta_{XX_\mathrm{L}X_\mathrm{R}} = \Delta_{X_\mathrm{L}X_\mathrm{R}}$ and $\Delta_{XX_\mathrm{R}} = \Delta_{X_\mathrm{R}}$.

Supplementary Note 4 To calculate the temporal correlations, we present a numerical model incorporating the energy levels along with the pumping and decay rates of important transitions. The model includes eight energy states $|G\rangle$, $|X_\mathrm{L}\rangle$, $|X_\mathrm{R}\rangle$, $|XX_\mathrm{L}\rangle$, $|X_\mathrm{L}X_\mathrm{R}\rangle$, $|XX_\mathrm{R}\rangle$, $|XX_\mathrm{L}X_\mathrm{R}\rangle$ and $|X_\mathrm{L}XX_\mathrm{R}\rangle$, as illustrated in Supplementary Figure 4. The three primary transitions scrutinized in

the main text are highlighted with thick coloured arrows. We limit our Hilbert space up to the triexciton level because the formation of higher states is unlikely at the applied pump powers and their effect on temporal characteristics of desired resonances is trivial.

The diagram plotted in Supplementary Figure 4 can be represented in an 8×8 time propagation matrix expanded on $\mathbf{V} = |G\ \ X_{\rm L}\ \ X_{\rm R}\ \ XX_{\rm L}\ \ X_{\rm L}X_{\rm R}\ \ XX_{\rm L}X_{\rm R}\ \ XX_{\rm R}\ \ X_{\rm L}XX_{\rm R}\rangle^{\rm T}$ where $d\mathbf{V}/dt = \mathbf{MV}$:

$$\mathbf{M} = \left(\begin{array}{cccc} -\mathrm{W}_{\rm x}^{\rm L} - \mathrm{W}_{\rm x}^{\rm R} & \Gamma_{\rm L} & \Gamma_{\rm R} & 0 \\ \mathrm{W}_{\rm x}^{\rm L} & -\mathrm{W}_{\rm xx}^{\rm L} - \mathrm{W}_{\rm x}^{\rm R} - \mathrm{W}_{\rm T} - \Gamma_{\rm L} & 0 & 2\Gamma_{\rm L} \\ \mathrm{W}_{\rm x}^{\rm R} & \mathrm{W}_{\rm T} & -\mathrm{W}_{\rm xx}^{\rm R} - \mathrm{W}_{\rm x}^{\rm L} - \Gamma_{\rm R} & 0 \\ 0 & \mathrm{W}_{\rm xx}^{\rm L} & 0 & -\mathrm{W}_{\rm x}^{\rm R} - 2\Gamma_{\rm L} - 2\mathrm{W}_{\rm T} \\ 0 & \mathrm{W}_{\rm x}^{\rm R} & \mathrm{W}_{\rm x}^{\rm L} & 2\mathrm{W}_{\rm T} \\ 0 & 0 & 0 & \mathrm{W}_{\rm x}^{\rm R} \\ 0 & 0 & \mathrm{W}_{\rm xx}^{\rm R} & 0 \\ 0 & 0 & 0 & 0 \end{array} \right.$$

$$
\left.\begin{matrix}
0 & 0 & 0 & 0 \\
\Gamma_{\mathrm{R}} & 0 & 0 & 0 \\
\Gamma_{\mathrm{L}} & 0 & 2\Gamma_{\mathrm{R}} & 0 \\
0 & \Gamma_{\mathrm{R}} & 0 & 0 \\
-\mathrm{W}_{\mathrm{T}} - \mathrm{W}_{\mathrm{xx}}^{\mathrm{L}} - \mathrm{W}_{\mathrm{xx}}^{\mathrm{R}} - \Gamma_{\mathrm{L}} - \Gamma_{\mathrm{R}} & 2\Gamma_{\mathrm{L}} & 0 & 2\Gamma_{\mathrm{R}} \\
\mathrm{W}_{\mathrm{xx}}^{\mathrm{L}} & -2\Gamma_{\mathrm{L}} - \Gamma_{\mathrm{R}} - 2\mathrm{W}_{\mathrm{T}} & 0 & 0 \\
\mathrm{W}_{\mathrm{T}} & 0 & -\mathrm{W}_{\mathrm{x}}^{\mathrm{L}} - 2\Gamma_{\mathrm{R}} & \Gamma_{\mathrm{L}} \\
\mathrm{W}_{\mathrm{xx}}^{\mathrm{R}} & 2\mathrm{W}_{\mathrm{T}} & \mathrm{W}_{\mathrm{x}}^{\mathrm{L}} & -2\Gamma_{\mathrm{R}} - \Gamma_{\mathrm{L}}
\end{matrix}\right) \quad (1)
$$

.

The time evolution of $\mathbf{V}$ is obtained by diagonalizing $\mathbf{M}$, $\mathbf{M} = \mathbf{P\Lambda P}^{-1}$, and $\mathbf{V}(t)$ is given by $\mathbf{V}(t) = \mathbf{U}(t)\mathbf{V}(0)$, where $\mathbf{U}(t)$ is the unitary time evolution operator $\mathbf{U}(t) = \mathbf{P}e^{\mathbf{\Lambda}t}\mathbf{P}^{-1}$. Here $\mathrm{W}_{\mathrm{x}}^{\mathrm{L}}$ ($\mathrm{W}_{\mathrm{x}}^{\mathrm{R}}$) is the pumping rate of the exciton in the smaller (larger) dot. We assume that when the s shell is already occupied with an exciton, the effective pumping rate to form the second exciton ($\mathrm{W}_{\mathrm{xx}}^{\mathrm{L}}$, $\mathrm{W}_{\mathrm{xx}}^{\mathrm{R}}$) is lower. Γ_{L} (Γ_{R}) here is the recombination rate of an exciton in the smaller (larger) quantum dot. To extract Γ_{L} and Γ_{R} from the time-resolved PL experiments, we averaged over the $X_{\mathrm{L}}X_{\mathrm{R}}$ and X_{R} lifetimes measured at eight increasing pump powers and used the following relations: $\Gamma_{\mathrm{R}} = \Gamma_{X_{\mathrm{R}}} = 0.58\ (\mathrm{ns})^{-1}$, $\Gamma_{\mathrm{L}} = \Gamma_{X_{\mathrm{L}}X_{\mathrm{R}}} - \Gamma_{\mathrm{R}} = 0.32\ (\mathrm{ns})^{-1}$ (see Supplementary Figure 5a). The applied excitation powers are the same as used in the main text for observing the variations in the bunching visibility. Although the intrinsic lifetime of an exciton commonly deviates from a mono-exponential fit in a cascade recombination, we employed the average exciton decay rates considering that their variations versus pump power were negligible ($\Delta\Gamma = \pm 0.05\ (\mathrm{ns})^{-1}$). We

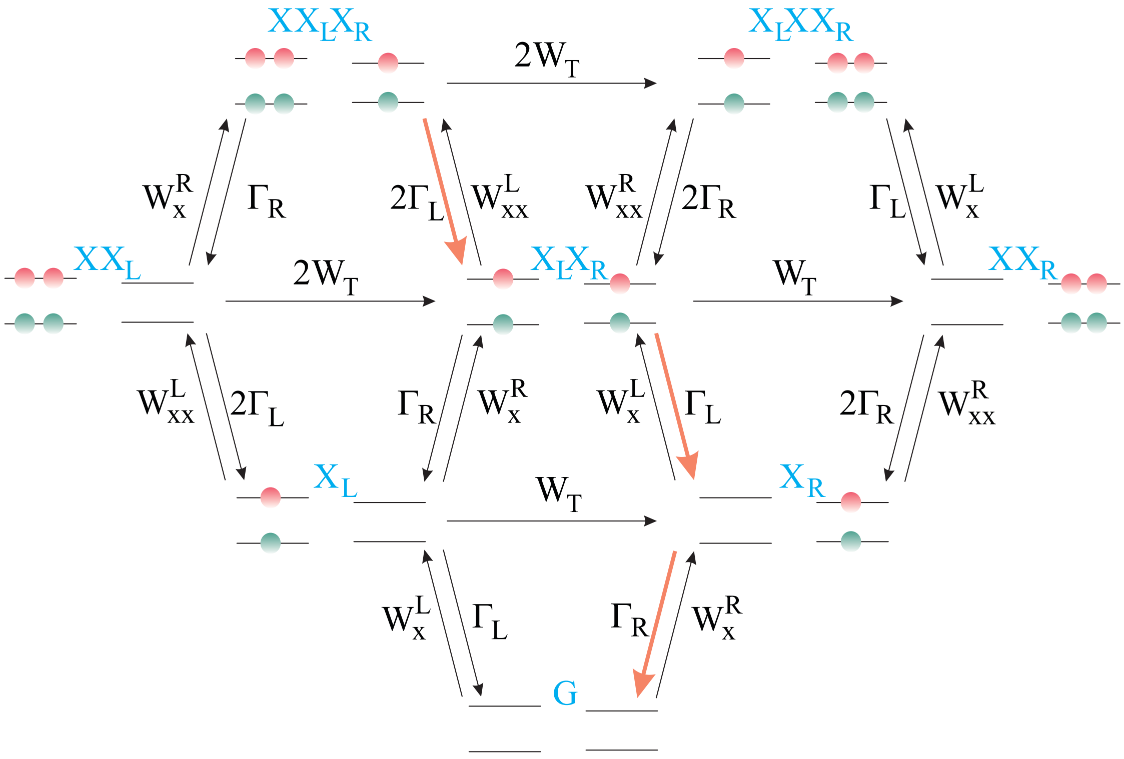

Supplementary Figure 4: Energy level structure of triexciton decay paths in a QDM Different configurations of exciton, biexciton and triexciton and the transition paths between them are plotted. Upward, downward and horizontal arrows denote excitation, decay and direct energy transfer rates, respectively. The red thick arrows show the $XX_{\mathrm{L}}X_{\mathrm{R}}$, $X_{\mathrm{L}}X_{\mathrm{R}}$ and X_{R} transitions in the main text.

also note that the $XX_{\mathrm{L}}X_{\mathrm{R}}$, $X_{\mathrm{L}}X_{\mathrm{R}}$ and X_{R} lifetimes comply with the ordering required for their cascaded nature,[4] i.e. $\Gamma_{XX_{\mathrm{L}}X_{\mathrm{R}}} > \Gamma_{X_{\mathrm{L}}X_{\mathrm{R}}} > \Gamma_{X_{\mathrm{R}}}$.

We accounted for the direct energy transfer (Förster transfer) of excitons among the two s shells as characterized by $\mathrm{W_T}$ in the diagram. Förster transfer originates from long-range interdot Coulomb interactions[5] and its rate depends on several parameters including energy difference of QDM s shells ($\Delta E = E_{X_{\mathrm{L}}} - E_{X_{\mathrm{R}}}$), interdot spacing and QDM symmetry.[6] Note that a sizeable interdot Coulomb coupling does not necessarily lead to the exciton Förster transfer, because the

transfer matrix element in QDMs with broken symmetry greatly depends on ΔE and phonon assisted mechanisms.[6] In practice, a direct energy transfer via the Förster coupling is likely between two molecular states in a relative resonance (< 5 meV).[6,7] An energy separation equivalent to ~ 70 meV between the s-shell resonances of the smaller and larger dots excludes such a direct tunneling mechanism ($\mathrm{W_T} \approx 0$) in our QDM. We note that the s shell in $\mathrm{QD_L}$ is located in the vicinity of upper shells in $\mathrm{QD_R}$ (perhaps d shells), and this may promote the nonresonant carrier tunneling from the d shells of $\mathrm{QD_R}$ into the s shell of $\mathrm{QD_L}$ via phonon-assisted coupling. Under such conditions, the effective pumping rate of the s shell in $\mathrm{QD_L}$, $\mathrm{W_x^L}$, will dynamically increase as compared to $\mathrm{W_x^R}$.

The important parameters used to model the measured cross correlations in the main text are compiled in Supplementary Table 1. The model is incapable of comprising the excitation power P_{exc} and the PL intensity of resonances. The influence of the incoherent pumping is reflected in the pump rate of transitions ($P_{\mathrm{exc}} \propto W_{\mathrm{P}}\hbar\omega_{\mathrm{P}}$ holds for a typical laser transition, however we repudiate such exact proportionality for quantum dot transitions). We found that $\mathrm{W_{xx}^L}$ or $\mathrm{W_{xx}^R}$ cannot grow fast and must be sufficiently smaller than $\mathrm{W_x^L}$ or $\mathrm{W_x^R}$, to precisely fit to the bunching peaks of $XX_{\mathrm{L}}X_{\mathrm{R}}$-$X_{\mathrm{L}}X_{\mathrm{R}}$ and $XX_{\mathrm{L}}X_{\mathrm{R}}$-$X_{\mathrm{R}}$ measured correlation. For simplicity, we presumed $\mathrm{W_{xx}^L} = \mathrm{W_{xx}^R}$ but their ratio can be similar to $\mathrm{W_x^L}/\mathrm{W_x^R}$. As inferred from Supplementary Table 1, $\mathrm{W_x^L}$ and $\mathrm{W_x^R}$ are enhanced linearly (except for low excitation powers around the $XX_{\mathrm{L}}X_{\mathrm{R}}$ onset), while $\mathrm{W_{xx}^L}$ and $\mathrm{W_{xx}^R}$ are increased linearly throughout the range. It is not feasible to pinpoint the exact values of pumping rates due to the variety of parameters involved in the actual experiment and the approximative nature of our model. The impact of detector was introduced into the correlations

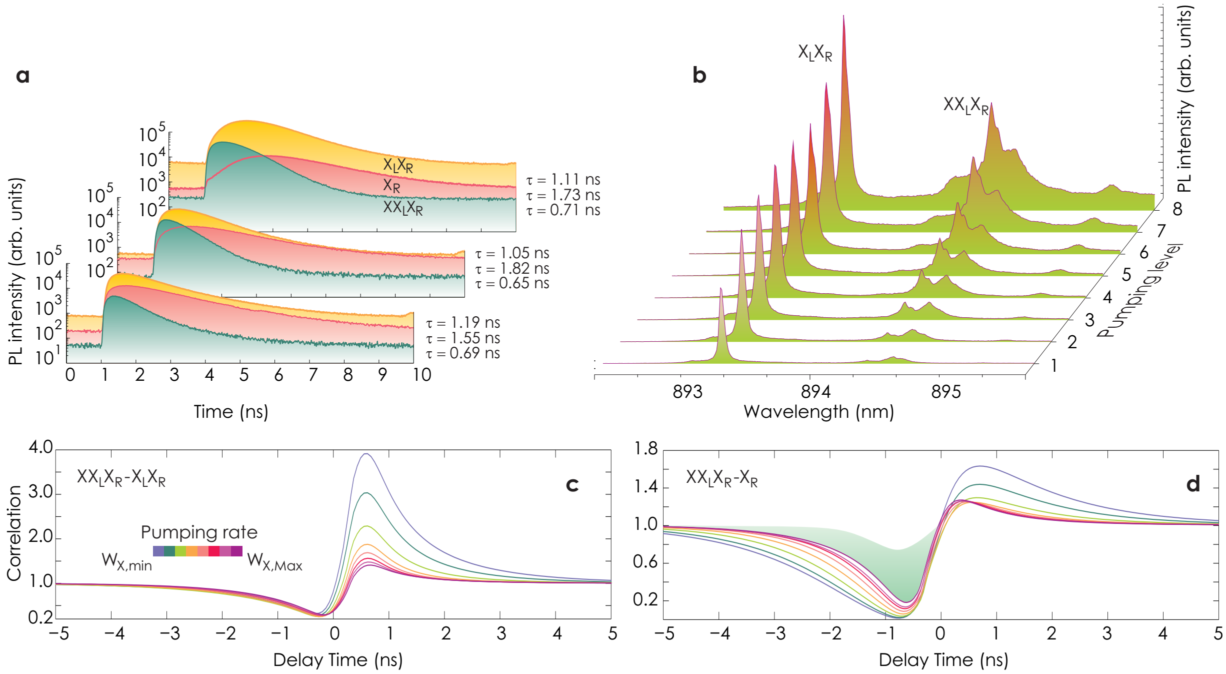

Supplementary Figure 5: Power dependent PL intensity and photon correlation visibility a) The time-resolved PL intensity of X_R, $X_\mathrm{L}X_\mathrm{R}$ and $XX_\mathrm{L}X_\mathrm{R}$ resonances displayed for the first, fourth and seventh spectra plotted in (b). The fitted lifetimes undergo an insignificant change even though elevating the excitation power leads the violation of the monoexponential fit. The output power of the pulsed laser is adjusted to reproduce the same spectra as seen in the cw mode in (b). The temporal resolution for the time-resolved measurement is set to 30 ps. b) The H.E. set spectra plotted for the eight increasing power densities starting from 220 $\mathrm{mW/mm^2}$. (c-d) The cross-correlations of $XX_\mathrm{L}X_\mathrm{R}$ resonance with $X_\mathrm{L}X_\mathrm{R}$ and X_R resonances calculated empirically using the time-propagation model. The curves are color-coded according to the excitation rates listed in Supplementary Table 1. The green-shaded area shows the difference in the antibunching dip between the modeling and experimental data due to the excessive background light collected by the multimode fibre.

Supplementary Table 1: Parameters used to produce the cross correlation curves in Supplementary Figure 5. $\mathrm{W}_{\mathrm{x}}^{\mathrm{L}}$, $\mathrm{W}_{\mathrm{x}}^{\mathrm{R}}$, $\mathrm{W}_{\mathrm{xx}}^{\mathrm{L}}$ and $\mathrm{W}_{\mathrm{xx}}^{\mathrm{R}}$ are chosen to fit the numerical curves to the experimental data, whereas $\Gamma_{X_{\mathrm{L}}X_{\mathrm{R}}}$ and $\Gamma_{X_{\mathrm{R}}}$ are measured experimentally.

Curve Num.	$\mathrm{W}_{\mathrm{x}}^{\mathrm{L}}$ (ns^{-1})	$\mathrm{W}_{\mathrm{x}}^{\mathrm{R}}$ (ns^{-1})	$\mathrm{W}_{\mathrm{xx}}^{\mathrm{L}}$ (ns^{-1})	$\mathrm{W}_{\mathrm{xx}}^{\mathrm{R}}$ (ns^{-1})	$\Gamma_{X_{\mathrm{L}}X_{\mathrm{R}}}$($\mathrm{ns}^{-1}$)	$\Gamma_{X_{\mathrm{R}}}$(ns^{-1})
1	0.97	0.24	0.20	0.20	0.838±0.05	0.643±0.05
2	1.18	0.33	0.21	0.21	0.898±0.05	0.633±0.05
3	1.57	0.55	0.23	0.23	0.934±0.05	0.555±0.05
4	2.15	0.84	0.26	0.26	0.950±0.05	0.549±0.05
5	2.89	1.20	0.30	0.30	0.907±0.05	0.563±0.05
6	3.77	1.64	0.31	0.31	0.897±0.05	0.561±0.05
7	4.57	2.03	0.32	0.32	0.900±0.05	0.576±0.05
8	5.74	2.85	0.34	0.34	0.879±0.05	0.586±0.05

through convolving them with a Guassian lineshape: $f = 1/\sqrt{2\pi\sigma_{\mathrm{D}}}\exp(-t^2/2\sigma_{\mathrm{D}}^2)$, where $\sigma_{\mathrm{D}} = 250$ ps is the APD temporal resolution.

The model given above merely accounts for the radiative excitation of neutral states and neglects the formation of charged particles that can affect the neutrality of quantum dots through nonradiative mechanisms. Moreover, the model does not directly incorporate the PL intensity of spectral lines. However, putting the accuracy of parameters aside, the model is qualitatively in a very good agreement with our experimental results approving the suppression of bunching visibility of a triple cascade in QDMs.

Supplementary Note 5 To further clarify the peculiarities seen in the triple coincidence histogram plotted in Fig. 3d in the main text, we simulated the three-photon correlations numerically. To comply with the observations, we assumed that $XX_{\mathrm{L}}X_{\mathrm{R}}$ serves as the Start signal while $X_{\mathrm{L}}X_{\mathrm{R}}$ and X_{R} function as Stop1 and Stop2, respectively. The temporal characteristics are treated consistent with the experiment. We model three different scenarios: first, three uncorrelated single photon sources of the same brightness and recombination energy as $XX_{\mathrm{L}}X_{\mathrm{R}}$, $X_{\mathrm{L}}X_{\mathrm{R}}$ and X_{R} are considered. Poissonian noise on every individual channel was taken into account to mimic a realistic counting experiment. The triple coincidence histogram is plotted in Supplementary Figure 6(a), where fully random contributions from the three photons form the background level (by average 319 counts). In the second (third) case, we correlate the measured twofold coincidences of $XX_{\mathrm{L}}X_{\mathrm{R}}$ and $X_{\mathrm{L}}X_{\mathrm{R}}$ (X_{R}) with uncorrelated X_{R} ($X_{\mathrm{L}}X_{\mathrm{R}}$) signal. The outcome reproduces the so-called "bunching walls" superimposed on top of the fully accidental events, see Supplementary Figure 6(b-c). These features are partially correlated and clearly observed as the dual-channel bunching along τ_{31} and τ_{21} axes in Fig. 3d in the main text. The third bunching wall in the experimental plot (not simulated here) originates from twofold correlations of $X_{\mathrm{L}}X_{\mathrm{R}}$ and X_{R} with $XX_{\mathrm{L}}X_{\mathrm{R}}$ being random. The threefold coincidence peak sits at the intersection of these three bunching features.

Supplementary Note 6 The diagram in Supplementary Figure 7 shows the spin configuration of triexciton, $\{|0_{\mathrm{XX,L}}, \mathrm{S_R}\rangle, |0_{\mathrm{XX,L}}, \mathrm{T_R}\rangle\}$, separated biexciton $\{|\mathrm{S_L}, \mathrm{S_R}\rangle, |\mathrm{T_L}, \mathrm{S_R}\rangle, |\mathrm{S_L}, \mathrm{T_R}\rangle, |\mathrm{T_L}, \mathrm{T_R}\rangle\}$ and exciton $\{|0_{\mathrm{L}}, \mathrm{S_R}\rangle, |0_{\mathrm{L}}, \mathrm{T_R}\rangle\}$ in the singlet and triplet basis, where $|0_{\mathrm{XX}}\rangle = |\uparrow\Downarrow\downarrow\Uparrow\rangle$, $|\mathrm{S}\rangle = 1/\sqrt{(}2)(|\downarrow\Uparrow\rangle - |\uparrow\Downarrow\rangle)$ and $|\mathrm{T}\rangle = 1/\sqrt{(}2)(|\downarrow\Uparrow\rangle + |\uparrow\Downarrow\rangle)$, $|0\rangle$ represents the ground states, and subscripts L and R denote the left and right quantum dot, respectively. The polarization of a

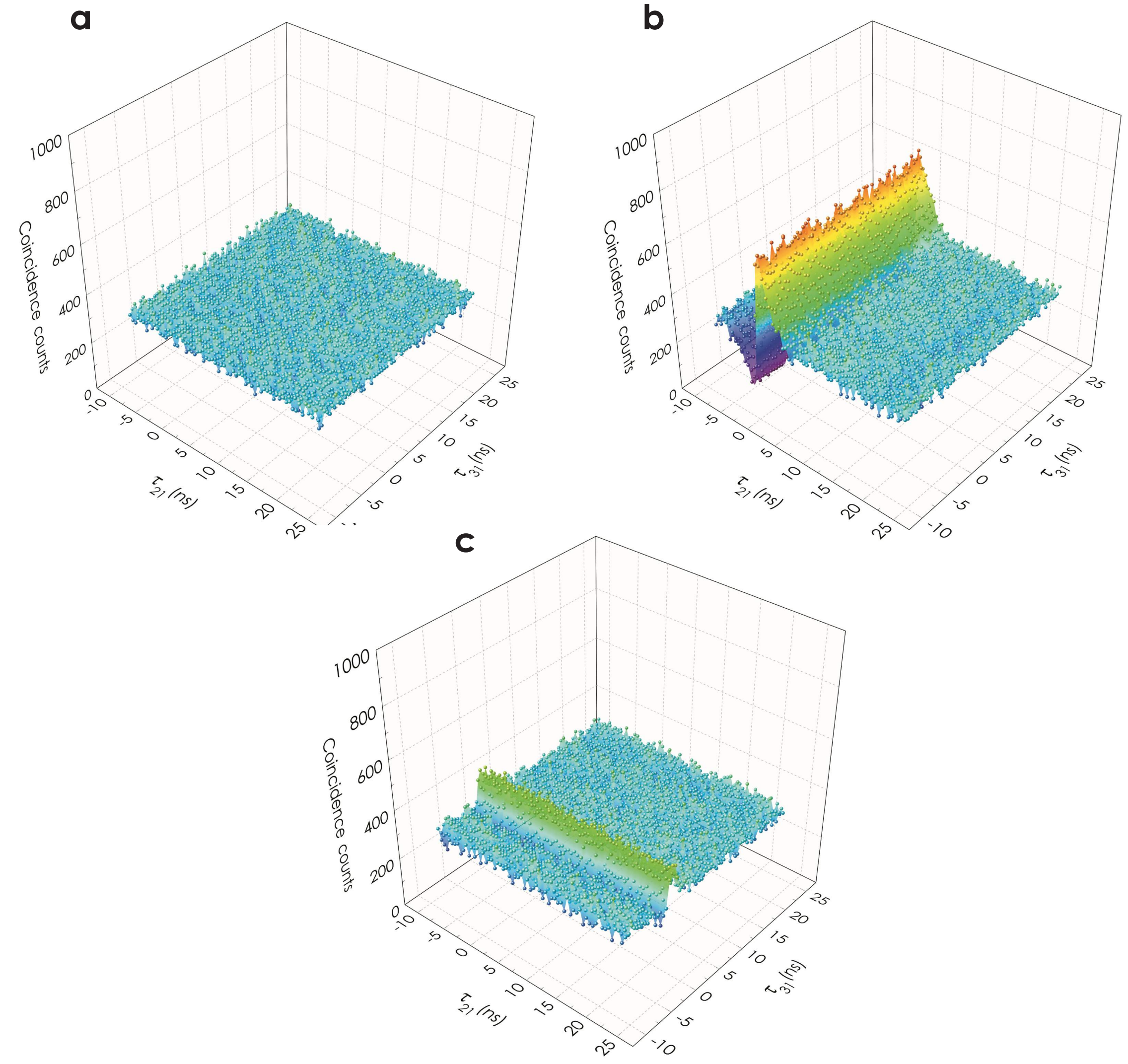

Supplementary Figure 6: Simulated triple coincidence counts a) background level made of fully random contributions from three uncorrelated photons. b) Correlated $XX_{\mathrm{L}}X_{\mathrm{R}}$ and $X_{\mathrm{L}}X_{\mathrm{R}}$ with random contribution from X_{R}. c) Correlated $XX_{\mathrm{L}}X_{\mathrm{R}}$ and X_{R} with random contribution from $X_{\mathrm{L}}X_{\mathrm{R}}$.

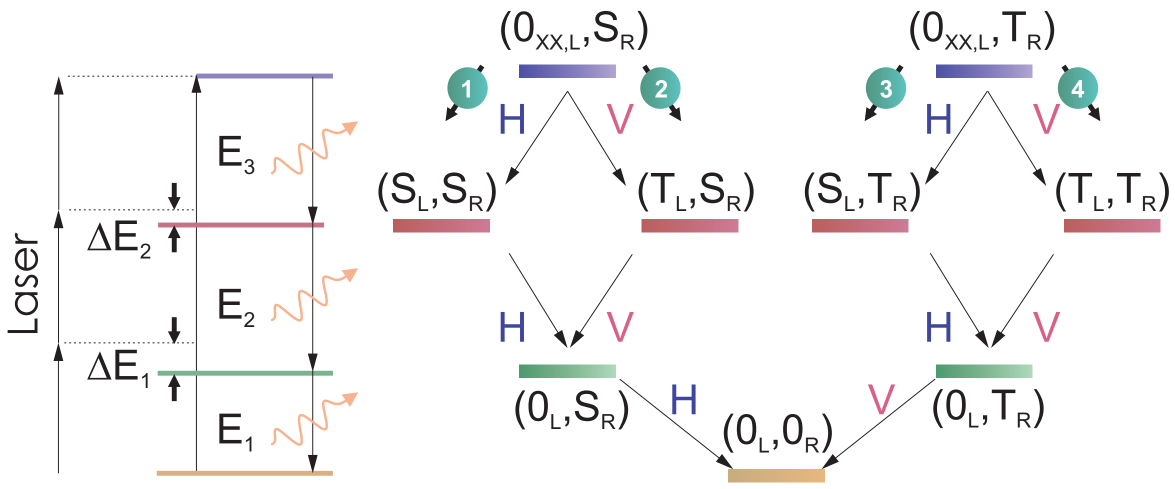

Supplementary Figure 7: Energy level diagram of the triexciton cascade in the singlet-triplet basis The triexciton can decay to the QDM ground state via four distinguished decay paths leaving linearly polarized photon triplets. The transition energies are distinct owing to the respective binding energies. Single and triplet states of each particle are either degenerate or split by the exchange-induced fines structure splitting (not shown here).

single photon emitted during the transition between each two states is labeled with H and V in the rectilinear basis. Depending on the pumping conditions, each of paths 1 to 4 can be used to generate time-bin entangled photon triplets through coherent excitation of either $|0_{\mathrm{XX,L}}, \mathrm{S_R}\rangle$ or $|0_{\mathrm{XX,L}}, \mathrm{T_R}\rangle$ and their subsequent transitions. We notice that the singlet-triplet configurations of each particle, $XX_{\mathrm{L}}X_{\mathrm{R}}$, $X_{\mathrm{L}}X_{\mathrm{R}}$ and X_{R}, are either degenerate or split by a relatively small spin exchange energy called fine structure splitting (FSS $< 100\ \mu$eV). The FSS in nanowire embedded quantum dots is theoretically predicted to reach values as small as zero,[8] which is also confirmed by recent polarization-dependent spectroscopy experiments.[9]

A fully coherent excitation of $|0_{\mathrm{XX,L}}, \mathrm{S_R}\rangle$ or $|0_{\mathrm{XX,L}}, \mathrm{T_R}\rangle$ states is, in principle, feasible by employing three different colored lasers in resonance with the transitions of interest, or using vir-

tual states. In the former case, a triexciton can be formed, e.g. by climbing path 2 via three resonant excitations and decay via path 1 to produce a well-defined $|\mathrm{HHH}\rangle$ three-photon state, while $|V\rangle$-polarized photons are filtered. The only experimental challenge here would be to block the laser scattering from the third transition $|0_\mathrm{L}, \mathrm{S_R}\rangle \rightarrow |0_\mathrm{L}, 0_\mathrm{R}\rangle$, as both beams are co-polarized. However this issue can be overcome by employing a quasi-resonant excitation of this particular transition combined with proper spectral filtering, or using ultrafast Pockels cells ($<$ 100 ps risetime) under resonant pumping to switch the laser polarization, followed by the polarization filtering.

In the latter case, a direct three-photon excitation up to $|0_\mathrm{XX,L}, \mathrm{S_R}\rangle$ is doable, however because of the large detuning between X_R and $XX_\mathrm{L}X_\mathrm{R}$ (or $X_\mathrm{L}X_\mathrm{R}$), X_R cannot be pumped efficiently ($\Delta E_2 \gg \Delta E_1$). Hence, combining the two-photon excitation of $XX_\mathrm{L}X_\mathrm{R}$ with the resonant (or quasi-resonant) pumping of X_R can be a viable solution. A schematic of the energy diagram for the above two methods is shown in Supplementary Figure 8a and b. Upon successful coherent excitation of $|0_\mathrm{XX,L}, \mathrm{S_R}\rangle$, or $|0_\mathrm{XX,L}, \mathrm{T_R}\rangle$, a two-photon time-bin entanglement scheme using analysing interferometers can be extended to realize entangled photon triplets.[10,11] A simplified schematic of the setup proposed for this experiment is depicted in Supplementary Figure 8. Supplementary Figure 8a illustrates the scheme where the two-photon resonant excitation of $XX_\mathrm{L}X_\mathrm{R}$ and a regular resonant (or quasi-resonant) pumping of X_R are combined, whereas in Figure 8b each transition is resonantly excited by a separate laser. In both setups the output pulse of each laser is split into two early and late pulses with a relative phase of $\phi_{\mathrm{p}i} = E_i \Delta t/\hbar$, where E_i is the energy of the QDM state in resonance and Δt is the time delay chosen longer than the coherence time of the photon. At sufficiently low pumping powers, the triexciton is formed either by the early or the late

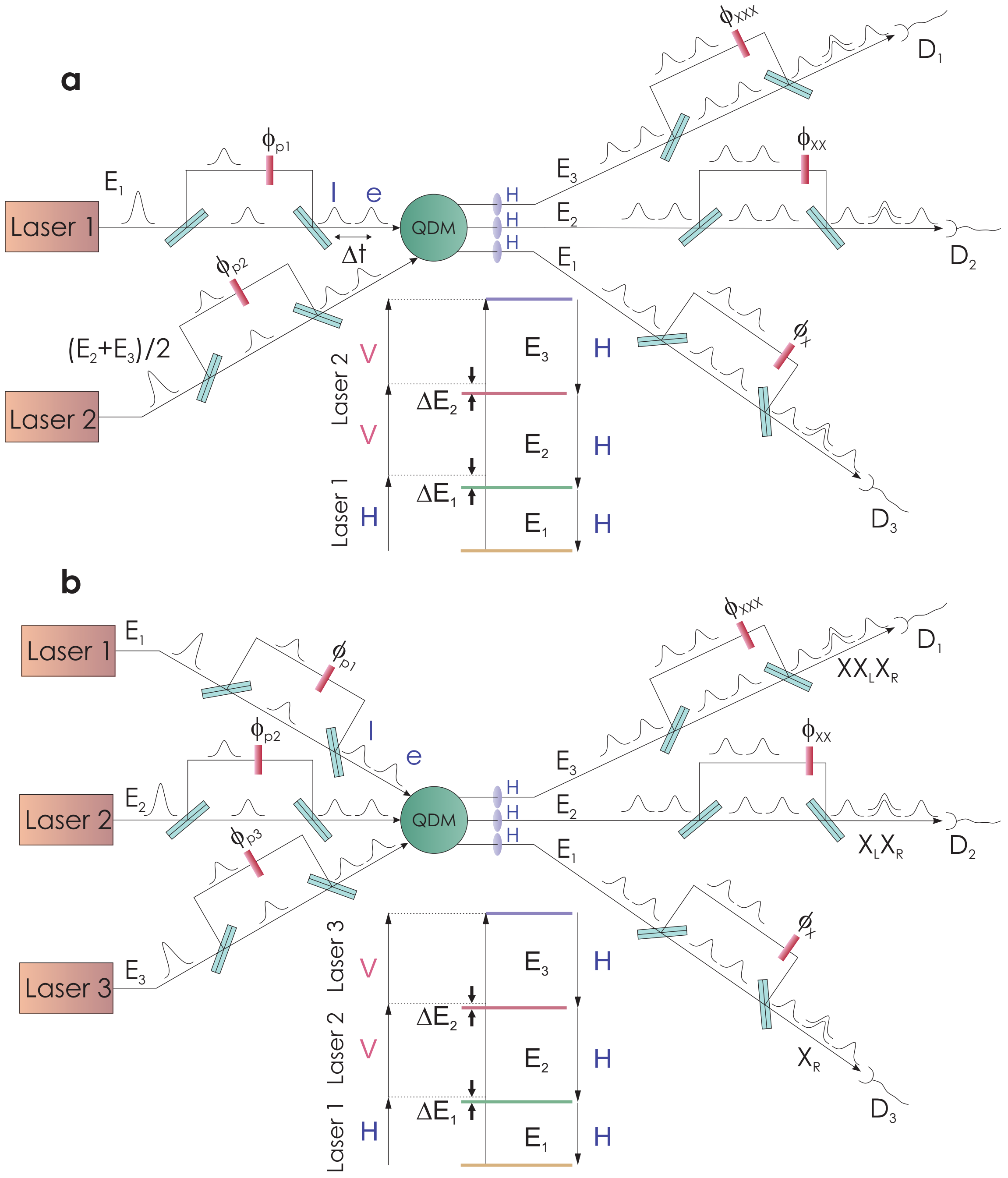

Supplementary Figure 8: Proposed setup for realization and measurment of time-bin entangled photon triplets

(a) Combination of two-photon and one-photon resonant excitation schemes. (b) Three-photon resonant excitation scheme. ΔE_1 can be slightly increased to facilitate the spectral filtering of the co-polarized laser beam.

pulse, followed by the emission of a triple photon cascade. The wavefunction of the three emitted photons can be represented as $|\psi\rangle = 1/\sqrt(2)(|e_1e_2e_3\rangle + e^{i\phi}|l_1l_2l_3\rangle)$, where the triple photons are in a superposition of being in the early or the late time bin. Here ϕ is the sum of the phases in the input interferometers. Three interferometers are required to control the phase of the entangled photons. Finally, a tomography experiment is performed by recording the coincidence between the outputs of the analysing interferometers.

Finally, we would like to discuss the maximum possible rate of photon triplet generation in the resonant excitation scheme. Upon incoherent pulsed excitation, the triplet creation rate is maximized once η_{C}, η_{D}, η_{G} and η_{F} (these efficiency values are defined within the main text) are all equal to unity, resulting a generation rate of 325 Hz (below 1 KHz). However, under the coherent excitation, the background noise and the amplitude of the side peaks will be drastically suppressed, and the triplet counts in the central peak is given by $\eta_{\mathrm{tot}} \times n_{\mathrm{P}} = \eta_{\mathrm{ex}}\eta_{\mathrm{D1}}\eta_{\mathrm{D2}}\eta_{\mathrm{D3}}\eta_{\mathrm{C}}^3\eta_{\mathrm{G}}^3\eta_{\mathrm{F}}^3 \times n_{\mathrm{P}}$ where η_{ex} is the excitation probability of triexciton reaching up to 90% with an optimized pulse length,[11] and n_{P} stands for the number of excitation pulses per second. In such a scheme, the amplitude of side peaks in the triple coincidence histogram is ideally zero and all the recorded counts in the central peak are considered as true photon triplets, leading to one registered triplet per excitation pulse. Consequently, with the efficiency values provided in the main text and the clock repetition rate n_{P} of 80 MHz, we obtain a photon triplet creation rate of about 17 KHz. With the assumption of ideal setup efficiency ($\eta_{\mathrm{D1}},\ \eta_{\mathrm{D2}},\ \eta_{\mathrm{D3}},\ \eta_{\mathrm{G}},\ \eta_{\mathrm{C}} = 1$) and an extraction efficiency equal to 0.46, the generation rate in the coherent pulsed excitation scheme can theoretically reach up to 7 MHz. However in practice, a non-ideal quantum efficiency and inevitable collection of background light

always limits this theoretical value as in our scheme.

Supplementary References

1. Khaetskii, A. V. & Nazarov, Y. V. Spin relaxation in semiconductor quantum dots. *Phys. Rev. B* **61**, 12639–12642 (2000).

2. Witek, B. J. *et al.* Measurement of the g-factor tensor in a quantum dot and disentanglement of exciton spins. *Phys. Rev. B* **84**, 195305–195311 (2011).

3. Bayer, M., Walck, S. N., Reinecke, T. L. & Forchel, A. Exciton binding energies and diamagnetic shifts in semiconductor quantum wires and quantum dots. *Phys. Rev. B* **57**, 6584–6591 (1998).

4. Persson, J., Aichele, T., Zwiller, V., Samuelson, L. & Benson, O. Three-photon cascade from single self-assembled InP quantum dots. *Phys. Rev. B* **69**, 233314–233317 (2004).

5. Govorov, A. O. Spin and energy transfer in nanocrystals without tunneling. *Phys. Rev. B* **68**, 075315–075320 (2003).

6. Govorov, A. O. Spin-förster transfer in optically excited quantum dots. *Phys. Rev. B* **71**, 155323–155331 (2005).

7. Rozbicki, E. & Machnikowski, P. Quantum kinetic theory of phonon-assisted excitation transfer in quantum dot molecules. *Phys. Rev. Lett.* **100**, 027401–027404 (2008).

8. Singh, R. & Bester, G. Nanowire quantum dots as an ideal source of entangled photon pairs. *Phys. Rev. Lett.* **103**, 063601–063604 (2009).

9. Versteegh, M. A. M. *et al.* Observation of strongly entangled photon pairs from a nanowire quantum dot. *Nature Communications* **5**, 5298–5303 (2014).

10. Jayakumar, H. *et al.* Time-bin entangled photons from a quantum dot. *Nature Communications* **5**, 4251–4255 (2014).

11. Huber, T. *et al.* Coherence and degree of time-bin entanglement from quantum dots. *Phys. Rev. B* **93**, 201301–201305 (2016).
